# Editorial: Relieving stress response in animals

**DOI:** 10.3389/fvets.2022.1098796

**Published:** 2022-12-14

**Authors:** Xiaojuan Wang, Jiaqing Hu, Lei Liu

**Affiliations:** Key Laboratory of Efficient Utilization of Non-grain Feed Resources (Co-construction by Ministry and Province), Ministry of Agriculture and Rural Affairs, Shandong Provincial Key Laboratory of Animal Biotechnology and Disease Control and Prevention, Department of Animal Science, Shandong Agricultural University, Taian, Shandong, China

**Keywords:** plant extracts, heat stress, feed additives, mitigative effect, response mechanism

As global warming continues unabated, the prevalence of heat stress in animals is projected to increase in terms of frequency, duration, and severity. Heat stress responses are now regarded as an expensive problem in the animals around the world. Heat stress could (1) activate hypothalamic-pituitary-adrenal (HPA) axis and increase glucocorticoids secretion, which could affect food intake *via* changing appetite peptides expression in the hypothalamus or gut, (2) affect food digestion and absorption *via* changing the expression of nutrient transporters and secretion of digestive enzymes, (3) affect the intestinal barrier *via* changing the intestinal microbiome and damaging the intestinal structure, (4) cause immune responses *via* increasing the level of inflammatory factors and inhibiting the level of immune factors, (5) induce severe oxidative stress *via* changing oxidative biomarkers levels, (6) affect the skeletal muscle development *via* changing the protein synthesis and decomposition, and (7) affect the lipid deposition *via* changing the glucolipid metabolism. The mitigation measures for heat stress affecting health are imperative.

Dihydromyricetin (a nature flavonoid compound extracted from *Ampelopsis grossedentata*) could protect dairy cow mammary epithelial cells against heat stress-induced injury through preventing oxidative stress, the imbalance of mitochondrial fission and fusion, which provides useful evidence that dihydromyricetin can be a promising therapeutic drug for protecting heat stress-induced mammary glands injury and mastitis (1). Astragalus polysaccharides have an effect on the serum hormones of heat-stressed dairy cows, and regulate the metabolism of heat-stressed dairy cows through glucose metabolism and amino acid metabolism pathways (2). Ginsenosides (ginseng extract) was found to be a suitable feed additive in animal nutrition to reduce the negative physiological effects caused by heat stress in intestinal barrier (3). Curcumin supplementation reversed the endoplasmic reticulum heat stress-mediated apoptosis in mice, indicating that curcumin supplementation alleviates physiological stress and cardiac damage caused by heat stress (4). Dietary addition of clove (*Syzygium aromaticum L*.) essential oil could significantly improve body weight gain and feed, and contribute to normalization of oxidative/nitrosative biomarkers in heat stressed broilers (5). Chlorogenic acid can ameliorate acute heat stress damage through suppressing inflammation and improved antioxidant capacity and cecal microbiota composition in mice (6).

Dietary addition of Radix bupleuri extract could maximum mitigate the negative effects of heat stress on body temperature and milk production in dairy cows. The improvement in milk production was probably not only due to the increased feed intake, but also to the direct mitigating effect of heat stress responses (decreased body temperature), which provides more energy for production rather than for homeothermy (7). Havlin et al. (8) reported that inclusion of citrus extracts in diet led to a higher proportion of Holstein cows lying down rather than standing, suggesting an improvement in comfort level. In addition, citrus extracts supplementation improved mammary health, as indicated by lower somatic cell count. Dietary supplementation of *B. vulgaris* root extract to quails reduced the detrimental effects of oxidative stress and lipid peroxidation resulting from Heat stress *via* activating the host defense system at the cellular level (9). The administration of *Rosa canina* extract attenuated reactive oxygen species levels and enhanced antioxidant defense in the hippocampus. Extract of *Rosa canina* attenuated the deleterious effect of Heat stress on cognition through its antioxidant properties and by enhancing synaptic function and plasticity (10). Supplementation with *Gingko biloba* extract before heat-stress exposure protected chicken myocardium from damage by increasing serum heat shock protein 70 (HSP70) from myocardial cells and cardiac microvascular endothelial cells and protected the microvascular system from adverse injury (Zhang et al.). Grape seed extract, a rich source of polyphenols, can attenuate the responses of jejunum epithelial cells induced by heat stress decreased protein concentrations of inflammatory factors and HSP70 (11). *Macleaya cordata* extract could alleviate heat stress-induced decline in growth performance by modulating blood biochemical markers and cecal flora composition in broilers (12). Turmeric or garlic extract supplementation could be an effective dietary supplementation to eliminate heat stress and improve health, oxidative capacity, and testicular functions of rabbit males (13). *Coptidis Rhizoma* could protect the brain against heat stress-mediated brain damage *via* amelioration of hyperthermia and neuroinflammation in mice, suggesting that fever-reducing *Coptidis Rhizoma* can attenuate thermal stress-induced neuropathology (14). Korean ginseng (*Panax ginseng Meyer*) suppressed the immune response upon heat stress and decreased the production of inflammatory cytokines in muscle and spleen and maintaining immune homeostasis (15).

In conclusion, Plant extracts have no residue, no resistance and no rest period, are considered as a green and safe feed additive, and will be continuously developed and applied in animal feeds to relieve heat stress.

## Author contributions

Writing: XW. Original draft preparation: JH. Funding acquisition, writing—review, and editing: LL. All authors have read and agreed to the published version.
